# Genetic and antigenic evolution of H9N2 subtype avian influenza virus in domestic chickens in southwestern China, 2013–2016

**DOI:** 10.1371/journal.pone.0171564

**Published:** 2017-02-03

**Authors:** Jing Xia, Jia-Qi Cui, Xiao He, Yue-Yue Liu, Ke-Chang Yao, San-Jie Cao, Xin-Feng Han, Yong Huang

**Affiliations:** College of Veterinary Medicine, Sichuan Agricultural University, Wenjiang, Chengdu, Sichuan, P. R. CHINA; University of California Davis, UNITED STATES

## Abstract

H9N2 avian influenza virus (AIV) has caused significant losses in chicken flocks throughout china in recent years. There is a limited understanding of the genetic and antigenic characteristics of the H9N2 virus isolated in chickens in southwestern China. In this study a total of 12 field strains were isolated from tissue samples from diseased chickens between 2013 and 2016. Phylogenetic analysis of the Hemagglutinin (HA) and Neuraminidase (NA) nucleotide sequences from the 12 field isolates and other reference strains showed that most of the isolates in the past four years could be clustered into a major branch (HA-branch A and NA-branch I) in the Clade h9.4.2 lineages. These sequences are accompanied by nine and seven new amino acids mutations in the HA and NA proteins, respectively, when compared with those previous to 2013. In addition, four new isolates were grouped into a minor branch (HA-branch B) in the Clade h9.4.2 lineages and two potential N-glycosylation sites were observed due to amino acid mutations in the HA protein. Three antigenic groups (1–3), which had low antigenic relatedness with two commonly used vaccines in China, were identified among the 12 isolates by antigenMap analysis. Immunoprotection testing showed that those two vaccines could efficiently prevent the shedding of branch A viruses but not branch B viruses. In conclusion, these results indicate the genotype of branch B may become epidemic in the next few years and that a new vaccine should be developed for the prevention of H9N2 AIV.

## Introduction

H9N2 avian influenza (AI) has become endemic in different types of terrestrial poultry in multiple countries on the Eurasian continent [1–3] in recent years. The spread of AI has resulted in significant economic losses due to reduced egg production and high mortality associated with co-infection with other respiratory pathogens [4, 5]. In China, H9N2 avian influenza virus (AIV) has been isolated in multiple animals including chicken, duck, quail, pheasant, partridge, pigeon, silky chicken, chukar, egret and swine [6, 7]. Furthermore, reassortment with other subtypes, including H6N1, H6N2, H5N1, H7N9 and H10N8, may cause the emergence of recombinant H9N2 strains which could also infect humans [6, 8–10].

Hemagglutinin (HA) protein is the receptor-binding and membrane fusion glycoprotein of AIV and the predominant inducer of a neutralizing antibody to virus infection [11]. Neuraminidase (NA) protein is also critical in the generation of progeny virions, which play a crucial role during the late stage of viral replication [12]. Both HA and NA proteins have a vital role in viral pathogenicity, antigenicity, and the host range of AIV [13]. Due to the incomplete proofreading mechanism of RNA polymerase in RNA virus, the generation of diversity in HA and NA genes may frequently occur and give rise to the emergence of new variant strains.

Genetic analysis showed that most of the H9N2 virus strains isolated since 2010 were clustered into genotype 57 in Clade h9.4.2 lineages (Y280-like). The genotype 57 strain, a reassortant of several strains, has become prevalent in vaccinated chickens in China and caused widespread outbreaks since 2010 [14–16]. However, since most of the commonly used vaccine strains, including A/chicken/Shandong/6/96 (SD696), A/chicken/Guangdong/SS/94 (SS) and A/chicken/Shandong/F/98 (F98) were isolated before 2000, the outbreak of H9N2 avian influenza in vaccinated commercial chickens is not surprising as a result of infections with field strains that antigenically differ from the vaccine strains [17, 18]. Since genetic and antigenic characteristics of H9N2 vary according to time, the characteristics of H9N2 strains from China since 2013 are not well known. In this study, the genetic and antigenic characteristics of H9N2 AIV strains circulating in commercial flocks in southwestern China in recent years were analyzed, and the protective efficacy of the currently used vaccine strains against H9N2 viruses of different antigenic groups were also evaluated. This result may provide critical insight for vaccine strain selection and vaccine development.

## Materials and methods

### Eggs and virus

Specific pathogen free (SPF) chicken embryos and one-day old SPF chickens were obtained from Beijing Merial Vital Laboratory Animal Technology Co., Ltd (Beijing, China). The 28-day old commercial Cobb broilers were obtained from Wenjiang Chia Tai Co., Ltd (Chengdu, China). The inactivated vaccine of SD696 and SS strains were selected for analysis of immunoprotection in this study. The inactivated oil vaccine of SD696 strain was obtained from the Qianyuanhao Biological Corporation Limited [Approval number: (2010)160132076, Beijing, China], and the inactivated oil vaccine of SS strain was obtained from the Guangdong Wens Dahuanong Biotechnology Co., Ltd [Approval number: (2011)190032080, Guangdong, China]. The inactivated antigen was also purchased from the same company.

### Sample collection and viral isolation

From 2013 to 2016, samples were collected from chicken farms in southwestern China including Si-chuan, Yun-nan, Gui-zhou, and Chong-qing. This collection was not a routine monitoring but from chicken farms with cases of suspected H9N2 AI which requiring a further laboratory diagnosis. The kidney, lung and trachea were collected together from dead chicken, or chickens with overt respiratory signs and/or reduction in egg production and euthanized by the local veterinarian. The collected tissues from one farm was referred as one sample. The samples were transported on dry ice to our lab and stored at—80°C prior to further processing. The samples were then homogenized in phosphate-buffered saline (PBS) containing 200 μg/mL penicillin and 100 μg/mL streptomycin in a ratio of 1:5–10. After centrifugating at 1,000 × g for 10 min at 4°C, the supernatants were filter sterilized with 0.22-μm filter membrane and inoculated into the allantoic cavities of 10-day-old SPF embryos (0.2 mL per embryo). The embryos were incubated at 37°C and examined twice daily for their viability. The allantois fluids were harvested after 48 h incubation and three blind passages were conducted [19]. The presence of H9N2 in tissue supernatants or allantois fluids were verified by reverse transcription-polymerase chain reaction (RT-PCR) analysis of the HA gene using the primers H9F (5ʹ-GGAAGAATCCTGAAGACTGA-3ʹ) and H9R (5ʹ-TCAAGCAGCACTAGCAATTC-3ʹ). The hemagglutination activity test was also used for the titration of the H9N2 virus in allantois fluids. The existence of other respiratory pathogens including Newcastle disease virus (NDV), infectious bronchitis virus (IBV) and infectious laryngotracheitis virus (ILTV) in tissue samples were verified by RT-PCR or PCR by following previously published methods [20–22]. Bacteria such as *Escherichia coli* and *Salmonella* were also isolated by blood agar culturing.

### Phylogenetic analysis of the HA and NA genes

Total RNA extraction and reverse transcription (RT) reaction was performed as previously reported [19]. PCR amplification of the HA gene was carried out using the primers HAF (5ʹ-TCTATCTGCTGCCATACCAACCC-3ʹ) and HAR (5ʹ-AGTAGAAACAAGGGTGTTTT TG–3ʹ). PCR amplification of the NA gene was carried out using the primers NAF (5ʹ-TGAATCCAAATCAGAAGATAATAGC-3ʹ) and NAR (5ʹ-CCCTAAAATTGCGAAAGCT-3ʹ). The cloning of the HA and NA gene was performed as previously reported [20]. The recombinant plasmids containing the target gene were sequenced by Shanghai Sanggong Biological Engineering Technology & Services Co., Ltd (Shanghai, China).

Nucleotide sequences of the HA and NA genes obtained from the H9N2 AIVs were aligned using the Editseq program in the Lasergene package (DNASTAR Inc., Madison, WI, USA) and compared to the sequences of other reference H9N2 isolates using the MegAlign program. The reference isolates included strains from the past ten years, strains from four primary lineages (Clade h9.1-h9.4), strains from two secondary lineages (Clade h9.4.1-h9.4.2), and three vaccine strains. A phylogenetic tree of the HA and NA genes was created using the neighbor-joining method in MEGA version 7.0.14. Bootstrap values were determined from 1000 replicates of the original data.

The potential N-linked glycosylation sites of HA and NA genes were predicted using the online software NetNGlyc 1.0 Server. Predictions were performed only on the Asn-Xaa-Ser/Thr sequons.

### Antigenic analysis the H9N2 isolates and commercial vaccine strains

To investigate the antigenic relationship between the 12 H9N2 strains and two commonly used vaccine strains, antiserum for each strain was generated. In brief, after being propagated in embryonated chicken eggs, concentration of the field isolates was adjusted to 10^7^ EID_50_/0.2 ml and inactivated by incubating them with 0.1% formalin at 20°C for 10 hours. The inactivated field isolates were then emulsified with oil adjuvant (Montanide ISA 70 SEPPIC, France) at a ratio of 3:7, and inoculated subcutaneously twice (at a 2-week interval) into three 6 week old SPF chickens (n = 3). Chickens were held in separate biosafety level 2 (BSL2) isolators in the Laboratory Animal Center of Sichuan Agricultural University (Ya’an, Sichuan, China) with ad-libitum access to feed and water and maintained under uniform standard management conditions. Antisera from vaccinated chickens were collected at 12 days after the final immunization and stored at -20°C.

The HI test was performed using a 1% chicken red blood cell suspension according to the Manual of Diagnostic Tests and Vaccines for Terrestrial Animals 2016 (OIE, http://www.oie.net). The HI titer was expressed as the reciprocal of the highest serum dilution in which hemagglutination was completely inhibited. An antigenic cartography was performed by using the program AntigenMap (http://sysbio.cvm.msstate.edu/AntigenMap), which uses matrix completion multidimensional scaling to map HI titers in two dimensions [23]. The detailed settings were set as follows: Low Reactor Threshold: 20; Normalization Method: N1; Temporal Model: No; Rank: 2; Number of Iterations: 2000. Antigenic map analysis can display the antigenic differences between viruses, such as viruses with high antigenic relevance cluster closely on the map, while viruses with low antigenic relevance stay far away from each other.

### Immune protection analysis of commercial vaccines against H9N2 isolates

To evaluate the protection efficacy of the commercial inactivated vaccines SD696 and SS against the representative H9N2 field isolates, three representative field strains A/chicken/Chongqing/LP/2015 (Group 1), A/chicken/Guizhou/QZ/2015 (Group 2) and A/chicken/Sichuan/LB/2013 (Group 3), which are located in different regions on the antigenMap, were chosen as the challenge virus. Virus re-isolation from the trachea was the main index to evaluate the protective rate of the vaccines.

Commercial 28-day-old Cobb broilers (n = 100) without HI titer in sera were randomly divided into ten groups (named A-J). Group A, B and C were subcutaneously injected with 0.3 mL of SD696 vaccine; group D, E and F were subcutaneously injected with 0.3 mL of SS vaccine; while group G, H, I, and J were unvaccinated. At 21 days post-immunization (d.p.i), chickens in group A, D and G were challenged with 2×10^6^ EID_50_ of A/chicken/Chongqing/LP/2015 strain in 0.2 mL by intravenous injection; chickens in B, E and H were challenged with 2×10^6^ EID_50_ of A/chicken/Guizhou/QZ/2015 strain and chickens in C, F and I were challenged with A/chicken/Sichuan/LB/2013 strain as the same way, respectively. Chickens in group J were mock infected with 0.2 mL PBS. The birds in each group were held in separate biosafety level 2+ (BSL2+) isolators under negative pressure in the Laboratory Animal center of Sichuan Agricultural University (Ya’an, Sichuan, China) with ad-libitum access to feed and water and maintained under uniform standard management conditions. Birds were monitored and recorded daily for appetite, activity, fecal output, conjunctivitis, cyanosis of the cumb, ruffled feathers and dyspnea.

Tracheal swabs from each group were sampled at 3, 5 and 7 days post-challenge (d.p.c) and placed in 2 mL PBS (pH 7.0–7.4) supplemented with 5% new-born calf serum (NCS) (Zhejiang tian-hang Biological technology stock Co., Ltd, Zhejiang, China). After filter sterilizing with a 0.22 μm filter membrane, 0.2 ml of each sample was inoculated into the allantoic cavity of 9- to 11-day-old SPF chicken embryos for virus re-isolation and examined twice daily for their viability. Each sample was injected into three eggs. The allantoic fluids were harvested after 72 h incubation and were tested with the HA test. The negative HA allantoic fluid was passaged one more time in SPF eggs. At 14 d.p.c, all remaining chickens were euthanized and dissected for pathological observation.

### Ethics statement

All animal experiment such as generation of antiserum from SPF chickens and immune protection tests of commercial vaccines were conducted complying with protocols approved by the Sichuan provincial Laboratory Animal Management Committee [Permit Number: XYXK (Sichuan) 2014–187] and the Ethics and Animal Welfare Committee (EAWC) of Sichuan Agricultural University. Humane endpoints were observed and utilized over the entire experimental period. Birds that were either unable or unwilling to eat and/or drink during the animal experiments and all the remaining birds at the end of animal experiments were euthanized immediately by cervical dislocation or by the administration of intravenous sodium pentobarbital (100 mg/kg) by a trained technician and approved by the EAWC.

## Results

### Viral isolation

A total of 71 clinical samples, including trachea, lung, and kidney, were collected from dead or diseased chickens displaying respiratory symptoms and/or reduction in egg production from chicken flocks located in different areas of southwestern China including Si-chuan, Yun-nan, Gui-zhou, and Chong-qing areas. Twelve H9N2 AIV strains were isolated. RT-PCR detection of the clinical samples with H9N2 virus showed that only one sample had co-infection of infectious bronchitis virus (IBV) (1/12, 8.3%). Bacterial isolation showed that *E*. *coli* or *Salmonella* were also found in the clinical samples (3/12, 25%). The case histories of the local strains are listed in Table 1.

**Table 1:** Information for the 12 H9N2 subtype AIVs in this study.

H9N2 strains	Production type	location	co-infection	HA Accession number	NA Accession number
A/chicken/Sichuan/LB/2013	Broiler	Sichuan (Ya’an)	Single	KX768851	KX768863
A/chicken/Sichuan/XJ/2014	Broiler	Sichuan (Xinjin)	Single	KX768852	KX768864
A/chicken/Sichuan/YX/2014	Broiler	Sichuan (Ya’an)	Single	KX768853	KX768865
A/chicken/Chongqing/LP/2015	Broiler	Chongqing(Liangping)	IB	KX768854	KX768866
A/chicken/Guizhou/QZ/2015	Broiler	Guizhou(Guiyang)	Single	KX768855	KX768867
A/chicken/Sichuan/SZQ60/2015	Broiler	Sichuan (Meishan)	Bacteria	KX768856	KX768868
A/chicken/Yunnan/A615/2015	Broiler	Yunnan(Kunming)	Bacteria	KX768857	KX768869
A/chicken/Yunnan/YNKM/2015	Broiler	Yunnan(Kunming)	Single	KX768858	KX768870
A/chicken/Chongqing/CQ/2016	Broiler	Chongqing	Bacteria	KX768859	KX768871
A/chicken/Sichuan/LFY/2016	Broiler	Sichuan(Yibin)	Single	KX768860	KX768872
A/chicken/Sichuan/LMC/2016	Broiler	Sichuan(Meishan)	Single	KX768861	KX768873
A/chicken/Sichuan/ZYL/2016	Broiler	Sichuan (Yibin)	Single	KX768862	KX768874

### Phylogenetic and molecular analysis of the HA gene

**Fig 1:**
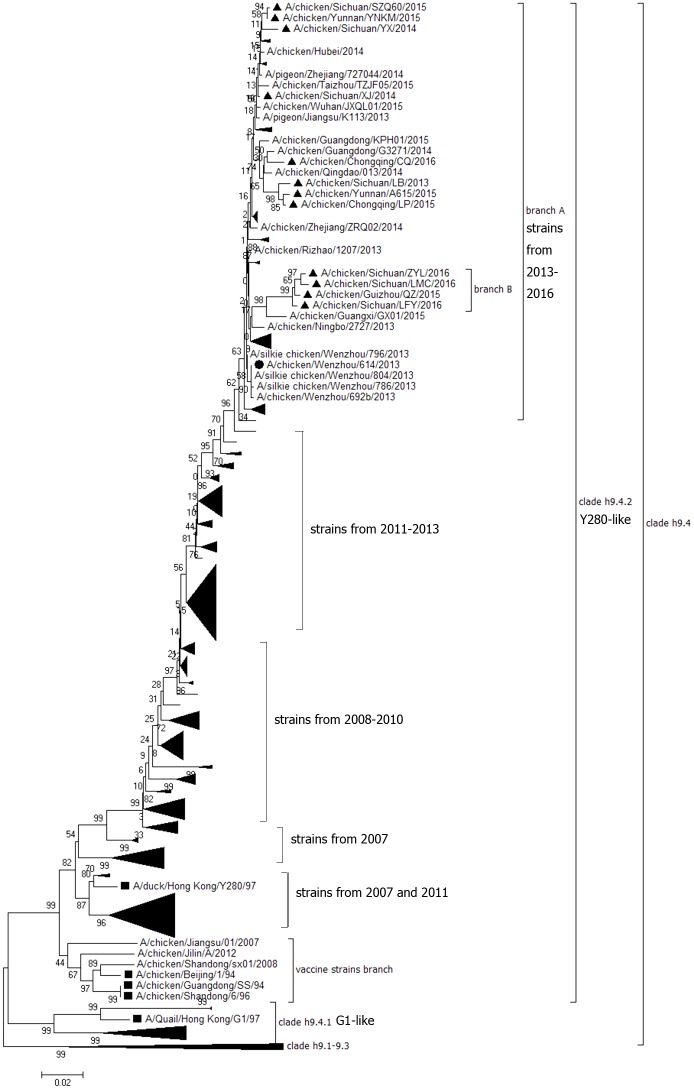
Phylogenetic analysis of the HA gene from 12 wild strains (filled triangles ▲) and 313 reference strains of H9N2 subtype AIVs. The sequences start at the AUG translation initiation codon and end at the stop codon. The phylogenetic tree was constructed using MEGA version 7.0.14 with the neighbor-joining method and 1000 bootstrap replicates. The filled square (■) represents the strains from each lineage and the filled circle (●) represents strains of genotype 57 identified by Pu J [28].

**Fig 2:**
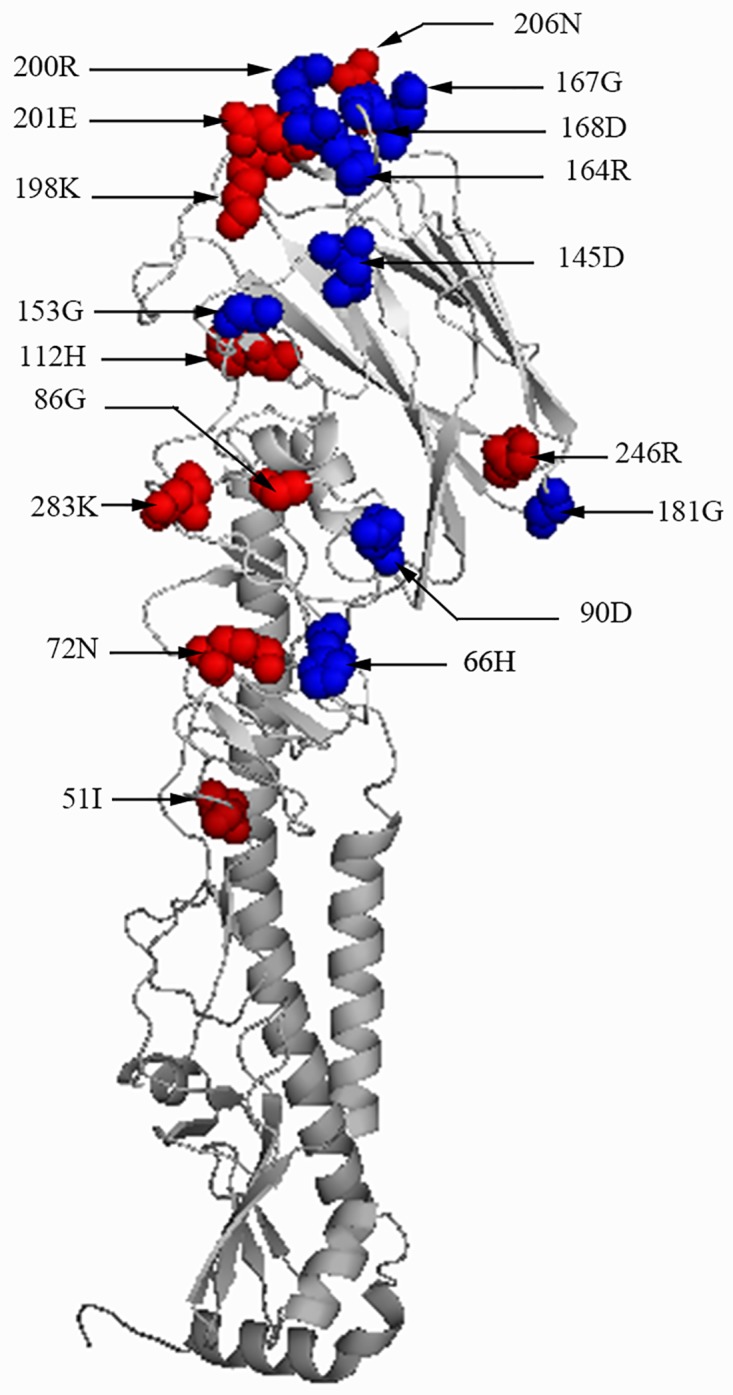
Secondary structure of the HA protein with mutations. The reference strain was A/chicken/Sichuan/LFY/2016. Mutations were shown as spheres representing the amino acid location. The mutations from branch A and B were shown as blue spheres and those from branch B were shown as red spheres.

HA genes from the 12 H9N2 AIV isolates were sequenced and submitted to GenBank under the accession numbers KX768851—KX768862. The open reading frame (ORF) of the HA gene from all 12 isolates were 1,683 bp long and encoded 560 amino acids. Phylogenetic analysis of the HA nucleotide sequences of the 12 field isolates and the other 319 reference strains including 311 field strains from China from the last 10 years (26 strains isolated in 2007, 20 strains in 2008, 34 strains in 2009, 26 strains in 2010, 39 strains in 2011, 60 strains in 2012, 47 strains in 2013, 39 strains in 2014 and 20 in 2015), five reference isolates from the lineages h9.1—h9.3, h9.4.1 and h9.4.2, and three vaccine strains was carried out (S1 Table). The results showed that Chinese strains in the h9.4.2 lineages isolated from 2013 to 2016 could be mainly clustered into two independent branches labeled A and B. Branch A included the major lineages and most of viruses isolated from 2013 to 2016. Branch B included new small lineages and isolates which had a unique HA protein (Fig 1). In this study, eight field isolates including A/chicken/Yunnan/YNKM/2015, A/chicken/Sichuan/SZQ60/2015, A/chicken/Sichuan/YX/2014, A/chicken/Sichuan/XJ/2014, A/chicken/Chongqing/CQ/2016, A/chicken/Sichuan/LB/2013, A/chicken/Chongqing/LP/2015 and A/chicken/Yunnan/A615/2015 were included in branch A, and four isolates including A/chicken/Sichuan/LFY/2016, A/chicken/Guizhou/QZ/2015, A/chicken/Sichuan/ZYL/2016 and A/chicken/Sichuan/LMC/2016 were included in branch B. Strains from branch A and B shared a high amino acid sequence similarity (95.4–97.1%), but shared a low identity (89.7–90.8%) with the commonly used vaccine strains F98, SS and SD696. Nine amino acids mutations including H66Q, G90E, S145D, D153G, Q164R, N167G, A168D, E181G, and T200R were observed in strains from branch A and B when compared with the Chinese isolates identified previous to 2013. All of these mutations were located at the head of the HA protein (Fig 2), and three of them including S145D, N167G and A168D were located at the critical HA antigenic sites identified previously [24–26]. Except for these nine mutations, ten additional amino acids mutations including L51I, T72N, D86G, E90D, N112H, T198K, D201E, T206N, K246R and R283K were observed in four strains from branch B when compared with the Chinese isolates identified previous to 2013 and seven of them were located near the RBS and left-right edge of receptor-binding pocket. Two new potential N-glycosylation sites (T72N and T206N) were observed in strains from branch B.

To validate whether these special mutations in strains from branch A and B could affect the advanced structure of the HA protein, the HA protein of strains from branch A and B and representative strain A/duck/Hong kong/Y280/97 were predicted with an online modelling tool (http://www.swissmodel.expasy.org/interactive) [27] and the predicted model were shown by the Pymol 0.99 software (created by Warren Delano). No differences were observed and the mutations were marked on the secondary structure of the HA protein (Fig 2).

The cleavage sites 333PSRSSR↓GL340 (H9 numbering), Receptor-binding sites (RBS) YWTNTLY (109, 161, 163, 191, 198, 202 and 203) and left-right edge of receptor-binding pocket 146GTSTA150 and 232NGLMGR237 were conserved among strains from branch A and B.

### Phylogenetic and molecular analysis of the NA gene

**Fig 3:**
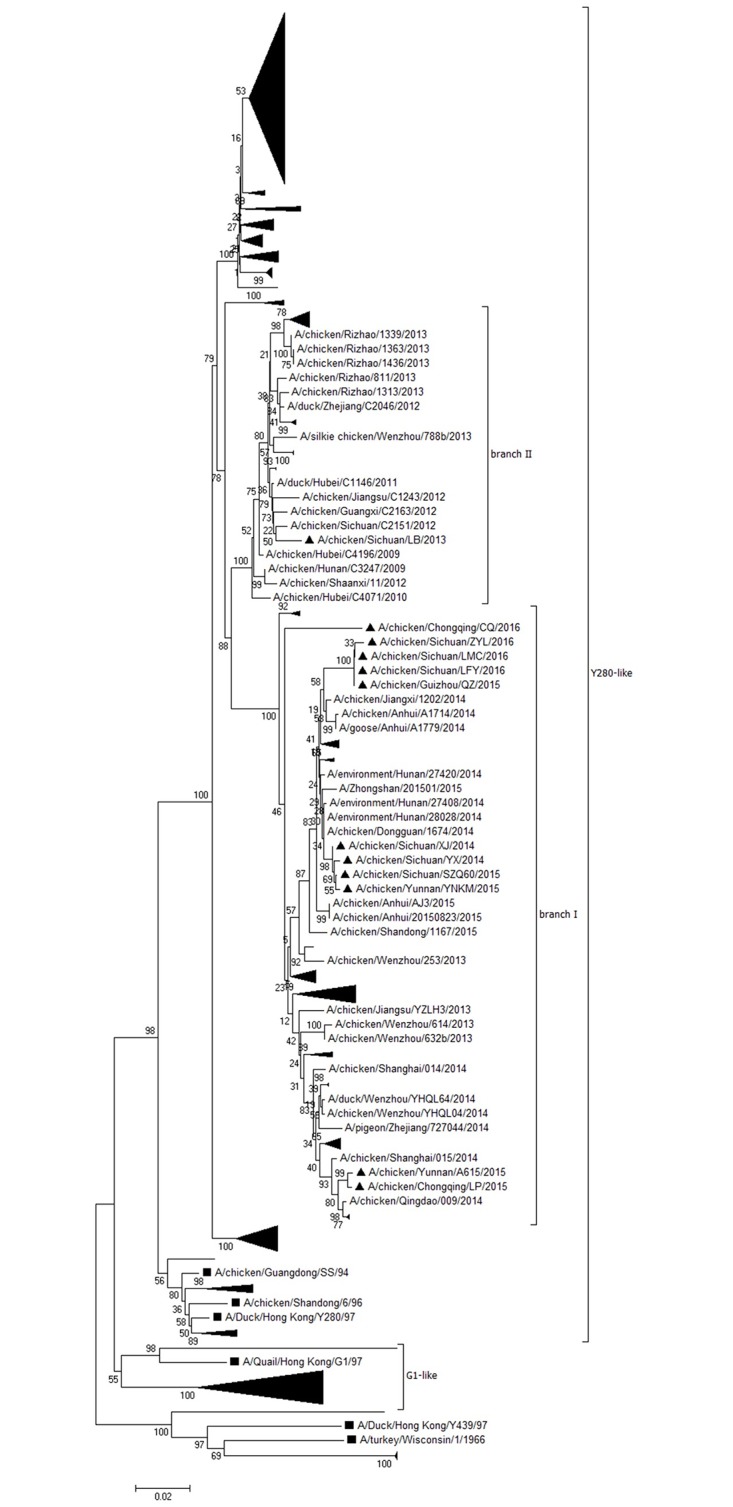
Phylogenetic analysis of the NA gene from 12 wild strains (filled triangles ▲) and 296 reference strains of H9N2 subtype AIVs. The sequences started at the AUG translation initiation codon and ending at the stop codon. The filled square (■) represents strains of each lineage.

The NA gene sequences of the isolates were submitted to GenBank under the accession numbers KX768863—KX768874. The full-length NA genes from 12 field strains were 1,401 bp and encoded 466 amino acids. Phylogenetic analysis of the NA nucleotide sequences from the 12 field isolates, 307 strains from China from the past 10 years (19 strains isolated in 2007, 23 strains in 2008, 30 strains in 2009, 30 strains in 2010, 50 strains in 2011, 50 strains in 2012, 49 strains in 2013, 42 strains in 2014 and 14 strains in 2015), five reference isolates and three vaccine strains were analyzed (S1 Table). Results showed that all 12 field isolates were grouped into Y280-like lineages and shared a high nucleotide identity (92.7–100%) with other Y280-like lineages strains isolated from 2013 to 2016. They also shared a low identity (89.7–93%) with commonly used vaccine strains F98, SS and SD696 as shown in Fig 3. The Chinese strains from h9.4.2 lineages isolated from 2013 to 2016 could be mainly grouped into two branches. Branch I was the major lineage and included most of the viruses isolated from 2014 to 2016, while the branch II strains included most of viruses isolated in 2013. For the 12 field strains isolated in this study, only one isolate (A/chicken/Sichuan/LB/2013) was grouped into branch II and the other 11 strains were grouped into branch I. Sequence comparison showed that seven new amino acid mutations (T10T, F22L, V51M, K72R, G124D, I251V and V299I) were observed in strains from branch I when compared with strains from branch II. Two (T10T and F22L) of them were located in non-polar transmembrane regions, two (V51M and K72R) were located in the stalk, and three (G124D, I251V and V299I) were located in the head of the NA protein.

Hemadsorbing sites (HBS, 366–373, 399–403 and 431–433, H9 numbering), active center (140–157) and antigenic determinants (153, 197–199, 328–336, 339–347, 367–370, 400–403 and 431–434) in the NA protein of two branch strains were also analyzed. For HBS, all sequences from NA of the branch I strains had a mutation at position 369 (D369G) when compared with branch II strains. For the active center, most of branch I strains were 140LKNKHSNGT (T/A) HDRTPHRT157 and the mutation was observed at position 149 (I149T/A) when compared with branch II. For the seven antigenic determinants in the NA protein, four of them (153T, 197DDK199, 339DPNNERGAP347, 431PQEP434) were relatively conserved in two groups, where the other three antigenic determinants including 328NDDSSSSSN336, 367IKNGS370 and 400SDDW403 had mutations when compared with Group II.

### Antigenic cross-reactivity analysis

**Fig 4:**
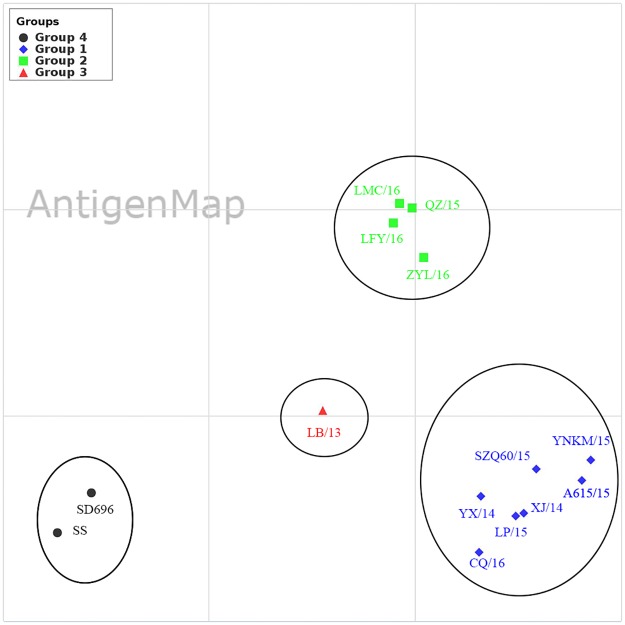
Antigen map of the 12 H9N2 AIV field isolates. Antigenic cartography representations of the HI data generated by using a panel of chicken antisera. One unit (grid) represents a 2-fold change in the HI assay results. Viruses in the same HI group were encircled in an oval. Virus designations were abbreviated as follows: YNKM/15 = A/chicken/Yunnan/YNKM/2015, SZQ60/15 = A/chicken/Sichuan/SZQ60/2015, YX/14 = A/chicken/Sichuan/YX/2014, XJ/14 = A/chicken/Sichuan/XJ/2014, CQ/16 = A/chicken/Chongqing/CQ/2016, LP/15 = A/chicken/Chongqing/LP/2015, A615/15 = A/chicken/Yunnan/A615/2015, LFY/16 = A/chicken/Sichuan/LFY/2016, QZ/15 = A/chicken/Guizhou/QZ/2015, ZYL/16 = A/chicken/Sichuan/ZYL/2016, LMC/16 = A/chicken/Sichuan/LMC/2016 and LB/13 = A/chicken/Sichuan/LB/2013.

Result of a reciprocal HI test showed that there is low reactivity between vaccine strains and recent viruses, while relatively high reactivity was observed between field strains (Table 2). Further antigenMap analysis showed that all 12 field isolates and two vaccine strains were divided into four independent antigenic groups (1–4) (Fig 4). The antigenicity differences between antigenic group-1 or group-2 strains and vaccine strains were more significant than those between group-3 strains and vaccine strains. Group-1 or Group-2 strains had at least 4-fold lower HI titers of the vaccine strains in reactions with the antisera to the vaccine strains. Group-1 contained seven strains which were grouped into branch A in the phylogenetic tree of the HA gene and branch I in the phylogenetic tree of NA (HA-branch A and NA-branch I); Group-2 included four strains which were grouped into branch B in the phylogenetic tree of the HA gene and branch I in the phylogenetic tree of the NA gene (HA-branch B and NA-branch I); Group-3 included one strain that was grouped into branch A in the phylogenetic tree of the HA gene and branch II in the phylogenetic tree of the NA gene (HA-branch A and NA-branch II); Group-4 included two vaccine strains SD696 and SS. These groups generally corresponded to the phylogenetic relationships of these viruses and were also correlated with the year of collection.

**Table 2:** Reciprocal HI titer of 12 H9N2 field isolates.

Virus	Antisera against different H9N2 field isolates											
	YX/14	A615/15	YNKM/15	LP/15	CQ/16	SZQ60/15	XJ/14	ZYL/16	LFY/16	LMC/16	QZ/15	LB/13
SD696	64	64	256	64	256	32	128	128	128	64	128	16
SS	128	16	256	128	128	16	128	64	128	32	128	16
YX/14	2048*	128	2048	512	512	512	1024	512	256	512	128	32
A615/15	1024	**512**	2048	512	512	1024	4096	1024	512	512	128	32
YNKM/15	1024	256	**4096**	512	1024	1024	2048	512	1024	512	256	32
LP/15	512	256	1024	**1024**	512	1024	1024	512	256	256	128	64
CQ/16	1024	512	512	512	**1024**	512	512	256	512	128	64	64
SZQ60/15	1024	256	512	512	1024	**1024**	2048	512	1024	256	256	32
XJ/14	512	256	1024	512	512	1024	**4096**	1024	512	128	128	32
ZYL/16	512	128	256	128	128	512	1024	**1024**	1024	1024	1024	32
LFY/16	512	128	512	128	64	512	512	1024	**1024**	1024	1024	32
LMC/16	512	128	512	128	64	512	512	1024	2048	**1024**	1024	32
QZ/15	512	512	1024	256	128	64	256	1024	2048	1024	**1024**	32
LB/13	1024	128	1024	64	256	64	256	256	512	256	64	**128**

Note:

* means the HI titer against the homologous strains, the abbreviation for each virus was the same as in Fig 4.

### Immune protection analysis

There were no unexpected deaths observed during the study. Three birds in group H and one in group I showed clinical signs such as lethargy, cough, dyspnea while no clinical signs were observed in the other groups. At 14 d.p.c, all animals were euthanized and dissected for pathological observation. Slight intestinal congestion and hemorrhage were observed in all of the groups except in group J, and slight tracheal congestion and hemorrhage was observed in A/chicken/Sichuan/LB/2013 challenged groups C, F and I.

For the chickens challenged by A/chicken/Chongqing/LP/2015 (Group-1 in antigenMap), percentage of chickens shedding the virus in the SD696 vaccine group, SS vaccine group and the control challenged group was 80%, 100% and 100% at 3 d.p.c, respectively. At 5 d.p.c., the percentage of virus shedding was 0%, 30% and 80% in the SD696 vaccine group, SS vaccine group and the control challenged group, respectively. There was no virus shedding in both of the two vaccine groups at 7 d.p.c., in contrast to the high virus re-isolation rate (80%) in the control group.

For the chickens challenged by A/chicken/Guizhou/QZ/2015 (Group-2 in antigenMap), the percentage of chickens shedding the virus in the SD696 vaccine group, SS vaccine group and the control challenged group at 3 d.p.c was 80%, 80% and 100%, respectively. At 5 d.p.c., the percentage of virus shedding was 50%, 80% and 100% in the SD696 vaccine group, SS vaccine group and the control challenged group, respectively. While there is still a relatively high virus re-isolation rate in SD696 (20%), SS (40%) and control group (60%) at d.p.c.

For the chickens challenged by A/chicken/Sichuan/LB/2013 (Group-3 in antigenMap), the percentage of chickens shedding the virus in the SD696 vaccine group, SS vaccine group and the control challenged group was 80%, 90% and 100% at 3 d.p.c, respectively. At 5 d.p.c., the percentage of virus shedding was 20%, 80% and 100% in the SD696 vaccine group, SS vaccine group and the control challenged group, respectively. There was no virus shedding in the SD696 vaccine group at 7 d.p.c, in contrasted to the relatively high virus re-isolation rate in the SS vaccine group (40%) and control group (60%) (Table 3).

**Table 3:** The clinical signs and virus re-isolation from chickens at different times post challenge.

Groups	Vaccine strains	Challenge strains	Re-isolation rate (%)			Respiratory symptoms ^a^, intestinal ^b^ and tracheal ^c^ congestion and hemorrhage
			3 d.p.c	5 d.p.c	7 d.p.c	
A	SD696	LP/15	80	0	0	b
B	SD696	QZ/15	80	50	20	b
C	SD696	LB/13	80	20	0	b, c
D	SS	LP/15	100	30	0	b
E	SS	QZ/15	80	80	40	b
F	SS	LB/13	90	80	40	b, c
G	-	LP/15	100	80	80	b
H	-	QZ/15	100	100	60	a, b
I	-	LB/13	100	100	60	a, b, c
J	-	PBS	0	0	0	-

## Discussion

H9N2 sequences were isolated recently in Chinese poultry farms and live-poultry markets [29]. Even though H9N2 is lowly pathogenic to chickens, it has played an important role in public health since 1998. Vaccination is an effective way to prevent AIVs outbreaks, but the vaccine should provide effective protection against the current field strains. The H9N2 vaccine strains currently used in China were selected from viruses isolated in the 1990s, but H9N2 strains isolated from 2009 to 2013 had undergone a significant antigenic drift from the vaccine strains (SD696 and F98) in China [14, 15, 26]. To isolate and identify the genetic and antigenic character of the current epidemic field strains it is very important to observe the evolutionary character of novel emerging variants and select the appropriate vaccine strains [30]. In this study, 12 H9N2 AIVs were isolated from vaccinated chickens from 2013 to 2016. Phylogenetic and antigenic analysis of those 12 isolates and other references were conducted, and an immune protection test of currently used inactivated vaccine against the representative strain of different antigenicity was also performed.

Previous studies on the phylogenetic analysis of the HA gene of H9N2 showed that Clade h9.4.1 lineages have been prevalent in China since the mid-1990s [15, 16, 31–33], and more than 74 genotypic groups in h9.4.2 lineages have been classified [28, 34]. In this study, phylogenetic analysis on the HA gene showed that all of the 12 isolates were correlated with genotype 57 (G57) of Clade h9.4.2 lineages strains. G57 strain was reassorted from six H9N2 strains in 2007 and has demonstrated improved adaptability to chickens and an increased host range such as domestic aquatic birds, wild birds, and swine [28]. All of the 12 isolates had a distant genetic relationship from the vaccine strains SD696 and SS, and had a L234 residue (H9 numbering) in the HA protein, which is also responsible for human-virus-like receptor specificity [35–37]. The cleavage site of the HA gene still contains the single and discontinuous basic amino acids (R) which conformed to the character of lowly pathogenic influenza viruses. Most of the analyzed Chinese strains isolated after 2013 formed a new major branch (Branch A) and a new minor branch (Branch B) in the phylogenetic tree. In addition, there are several amino acid site mutations in the HA protein from branch B, and most of the mutations were located near the RBS and left-right edge of the receptor-binding pocket which suggests the HA-Group B isolates may have a wider host range [28]. In addition, it was interesting to observe that the branch B strains had two new potential N-glycosylation sites in the HA protein. HA glycosylation can mask antigenic epitopes and therefore is an important process in the generation of escape mutants [38]. Sun and coworkers [39] showed that the increase in glycosylation site numbers mainly occurred with high frequency in the early stages of evolution of the influenza virus. The increase of potential N-glycosylation sites in the HA protein of Branch B strains implied that the genetic and antigenic evolution of Branch B strains should be paid more and more attention.

Phylogenetic analysis on the NA gene showed that all new isolates were grouped into Y280-like lineages similar to other research reported from China in recent years [15, 40]. Additionally, the NA protein of strains from the previous four years had several mutations and formed a new branch (branch I) when compared with strains previous to 2012. The mutations were located in the non-polar transmembrane region, stalk and head of the NA protein, but the function of these mutations was not clear.

The antigenic drift of H9N2 AIVs continues to occur in China [14, 41]. Since the country-wide administration of commercially inactivated H9N2 vaccines in chickens, the immunological pressure may have contributed to the antigenic drift of field strains. Reciprocal HI testing showed that all 12 field isolates had a low antigenic reactivity with vaccine strains SD696 and SS, which was further verified by the antigenMap analysis. A previous report [41] showed that the antigenic drift of influenza virus is not continuous but punctuated, and antigenically homogenous clusters of strains predominate for an average of three years. In this study strains from the adjacent year often clustered together in the antigenic map. The antigenic drift of influenza A virus was characterized by a complex interplay between frequent reassortment and periodic selective sweeps [41, 42]. In addition, strains in the same branch could also be diverted into different antigenic groups. For instance, A/chicken/Chongqing/LP/2015 and A/chicken/Sichuan/LB/2013 both belong to branch A but fall into antigenic group 1 and 3, respectively. This may be due to the fact that some mutations in HA may exert a disproportionately large effect on the antigenic type, whereas others are “hitchhikers” with no phenotypic effect [42].

The protection efficiency of vaccine SD696 against the H9N2 field strains in China has been previously studied and results showed that the antigenicity of most field isolates was distinctly different from vaccine strains [18, 38]. It is worth noting that the commonly used vaccine in China could not efficiently prevent the shedding of A/chicken/Guizhou/QZ/2015 strain from branch B viruses. We speculate that the branch B strains may have the potential to cause widespread outbreaks in next few years.

In conclusion, we have demonstrated that the genetic and antigenic characteristics of H9N2 AIVs isolated from southwestern China in recent years have undergone significant changes from the vaccine strains (SD696 and SS) and field isolates previous to 2013, and strains generated after 2013 formed a different genetic branch and antigenic profiles. Vaccine strains that have a good antigenic match with prevailing strains and which are broadly cross-reactive should be applied in the field.

## Supporting information

S1 Table The information of HA and NA gene of H9N2 reference strains in China from 2007 through 2015. (XLSX)
